# Electrospinning-Based Biosensors for Health Monitoring

**DOI:** 10.3390/bios12100876

**Published:** 2022-10-15

**Authors:** Guojing Ji, Zhou Chen, Hui Li, Desire Emefa Awuye, Mengdi Guan, Yingbao Zhu

**Affiliations:** 1 School of Mechanical and Power Engineering, Nanjing Tech University, Nanjing 211800, China; 2 Wuhu Innovation New Materials Co., Ltd., Wuhu 241080, China; 3 Department of Minerals and Materials Engineering, University of Mines and Technology, Tarkwa 03123, Ghana

**Keywords:** electrospinning, health monitoring, biosensor

## Abstract

In recent years, many different biosensors are being used to monitor physical health. Electrospun nanofiber materials have the advantages of high specific surface area, large porosity and simple operation. These properties play a vital role in biosensors. However, the mechanical properties of electrospun nanofibers are poor relative to other techniques of nanofiber production. At the same time, the organic solvents used in electrospinning are generally toxic and expensive. Meanwhile, the excellent performance of electrospun nanofibers brings about higher levels of sensitivity and detection range of biosensors. This paper summarizes the principle and application of electrospinning technology in biosensors and its comparison with other technologies.

## 1. Introduction

Globally, it has been observed that improvement in standards of living has also caused the rise of diseases. Biosensors that can monitor our physical health in real time are particularly important. Electrospinning technology has developed rapidly in recent years, and there are several nanofiber membranes that can be used in biosensors. The biosensor has high biocompatibility, good flexibility, remarkable mechanical properties and high sensitivity. For example, biosensors can assess the activity of enzymes in the body to determine the health of stem cells [1]. In recent years, such biosensors and bioelectronics devices that can monitor human health have developed rapidly. However, poor stability and short life span are the main problems in the current development of biosensors [2].

**Figure 1:**
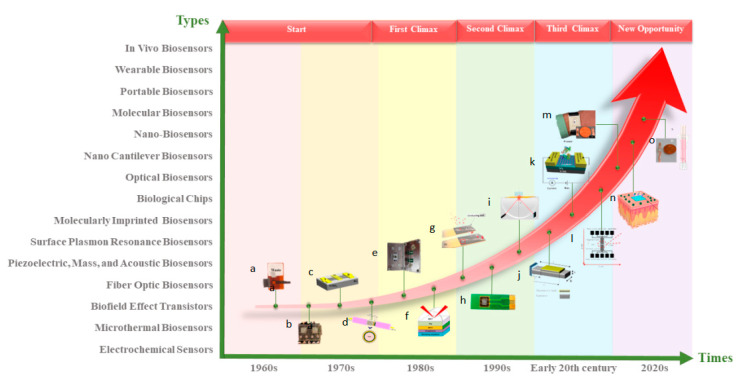
The main development history of biosensors: (**a**) electrochemical biosensors [6]; (**b**) microthermal biosensors [7]; (**c**) biofield effect transistors [8]; (**d**) fiber-optic biosensors [9]; (**e**) acoustic biosensors [10]; (**f**) surface plasmon resonance biosensors [11]; (**g**) molecularly imprinted biosensors [12]; (**h**) biological chips [13]; (**i**) optic biosensors [14]; (**j**) nano cantilever biosensors [15]; (**k**) nano-biosensors [16]; (**l**) molecular biosensors [17]; (**m**) portable biosensors [18]; (**n**) wearable biosensors [19]; (**o**) in vivo biosensors [20].

With the continuous advancement of current technology, there are many manufacturing methods for nanofiber membranes, with electrospinning being the easiest and relatively low-cost method. The nanofiber membrane made by electrospinning can form a typical network structure with high surface area and high porosity [3]. The process parameters can be modified to change the morphology and performance of the fiber membrane [4]. Due to these outstanding features, electrospun nanofiber membranes are more applicable in biosensors [5]. Figure 1 shows the main development history of biosensors. This paper reviews the principles and applications of electrospinning in biosensors.

## 2. Electrospinning

### 2.1. Principle of Electrospinning

**Figure 2:**
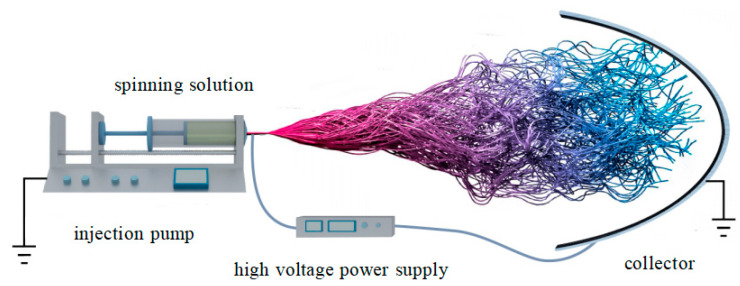
Schematic diagram of electrospinning work [22,23].

Electrospinning is a high-throughput technology that can produce nanofiber membranes with controllable fiber orientation [21]. In the whole working process, the Taylor cone formed by the solution is stretched by the electrostatic force to form a jet. When the jet is flying in the air, the solvent evaporates and the final solute is deposited on the receiving plate to form nanofibers. Figure 2 is a schematic diagram of the working principle of electrospinning [22,23]. The whole electrospinning equipment mainly includes three parts: a high voltage supply device, an injector and a receiving board. However, different requirements for nanofibers may lead to changes in one or several pieces of equipment. The change of the equipment is only to get the nanofibers needed while maintaining the working principle.

The influencing factors of electrospinning mainly include: solution concentration and viscosity, voltage intensity, receiving distance, air humidity and temperature. The viscosity and concentration of the solution play an important role in the morphology and performance of the fiber [24]. Voltage and receiving distance directly affect the thickness of the fiber [25]. The humidity and temperature of the air affect the volatilization of the solvent during the jet flight, which ultimately affects the formation of the fibers [26].

### 2.2. Types of Nanofibers

**Figure 3:**
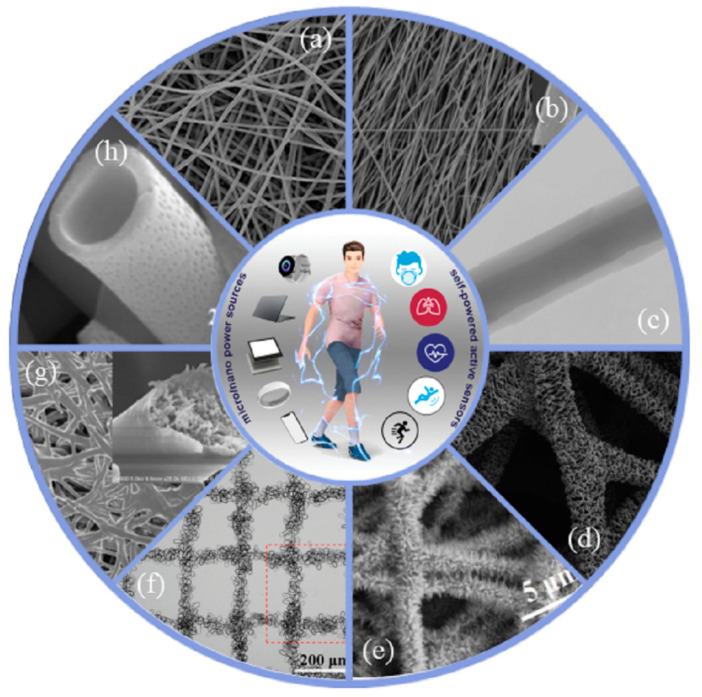
Morphological characteristics of different kinds of nanofibers: (**a**) randomly distributed nanofibers [29]; (**b**) aligned nanofibers [30]; (**c**) core-shell nanofibers [31]; (**d**) multipace nanofibers [32]; (**e**) pine-needle nanofibers [33]; (**f**) patterned nanofibers [34]; (**g**) cobweb nanofibers [35]; (**h**) hollow nanofibers [36].

Different parameters and methods of electrospinning lead to different morphology of nanofibers. For example, core-shell nanofibers can be obtained by coaxial electrospinning. Different kinds of nanofibers have different characteristics such as oriented nanofibers with high axial mechanical properties and good dimensional stability. High porosity and large specific surface area are key properties that contribute to a successful biosensor, hence the wide use of electrospun nanofibers [27]. Meanwhile, biosensors based on nanofibers have the advantages of high responsiveness, high sensitivity, wide detection object and cost effectiveness [28]. Figure 3 shows the morphological characteristics of different kinds of nanofibers.

### 2.3. Preparation and Characteristics of Nanofibers

The types of nanofibers are described above and the preparation techniques used for different nanofibers are also different. The preparation technology and characteristics of different fibers are described in detail below.

1. Randomly distributed nanofibers: The preparation of such fibers is the simplest form by direct drawing of the instabilities of the jet. This results in a relatively small range of applications and poor mechanical properties for such fibers. However, the advantage is that the preparation is simple and there is no complicated process.
2. Aligned nanofibers: This fiber is obtained by suppressing the instability of the jet on the basis of randomly distributed nanofibers. This makes the fibers have the advantages of high axial mechanical strength, good dimensional stability and high application value in tissue engineering, composite reinforcement, electrical and optical fields. However, due to the different methods used, the collection speed may be slow and the amount of fibers is relatively small.
3. Core-shell nanofibers: Fibers of this structure are mostly obtained by coaxial electrospinning devices. Core-shell structured nanofibers solve the problem that some materials are not spinnable. Core-shell structured nanofibers solve the problem that some materials are not spinnable. This also makes this fiber widely used in biomedical fields such as drug release systems, tissue engineering scaffolds, drug-loaded medical dressings and sutures.
4. Multispace nanofibers: The preparation of such fibers is mostly used to induce phase separation. For example, solvent evaporation and heating can induce phase separation to form multispace nanofibers. This fiber is characterized by a substantial increase in the specific surface area of the fiber.
5. Pine-needle nanofibers: Such fibers are formed based on randomly distributed nanofibers. Then, other materials are generally grown on randomly distributed fibers by hydrothermal method.
6. Patterned nanofibers: This fiber is obtained by changing the shape, movement mode and material of the collecting device. Its physical and chemical properties are basically the same as those of disordered fibers.
7. Cobweb nanofibers: This fiber is a two-dimensional mesh fiber membrane material with ultrafine electrospun fibers as a scaffold. It has the advantages of large specific surface area, good adsorption and stable mechanical properties.
8. Hollow nanofibers: This fiber is obtained on the basis of the core-shell structure fiber. Such fibers are generally obtained by coaxial electrospinning with a soluble or volatile substance as the core layer and a polymer solution as the shell layer and then removing the core layer by dissolving or heating. The disadvantage is that the production efficiency is relatively low.

## 3. The Principle and Characteristics of Biosensors

### 3.1. Working Principle

**Figure 4:**
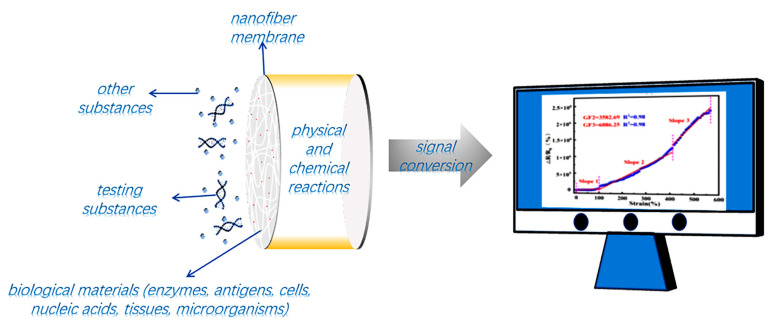
Fundamentals of biosensors.

A biosensor is mainly composed of a biosensor and signal transducer. The main function of the biosensor is to select the substance to be measured. The main function of signal transducers is to convert the chemical effects generated by the interaction between the biological components and measured substances into electrical signals that can be considered as output [37]. Thus, it works in two steps. Firstly, biological substances with molecular recognition function are coated and fixed on the carrier by technical means (such as electrospinning) to form a functional membrane. Secondly, in the working process, the biological substances react with the measured substances and then carry out signal conversion through the signal transmitter [38]. Figure 4 shows the main schematic diagram of the biosensor.

Electrospinning is also widely used in the field of biosensors. This is because the nanofiber film obtained by electrospinning has a high specific surface area. This enables more biological elements to be loaded on the fiber surface, which greatly improves its sensitivity. This is why electrospinning technology has been widely used in the field of biosensors in recent years. Table 1 lists several common biological elements and loading methods.

### 3.2. Characteristics of Biosensors

There are four main characteristics of biosensors (selectivity, sensitivity, reproducibility and stability) [44]. Selectivity is an important characteristic to be considered when biosensors select receptors. Only when biological elements interact with receptors can they produce positive effects. Since general biological samples contain a variety of biological information, selectivity is particularly important for sensors. Sensitivity is one of the important indicators to measure the quality of sensors. High-sensitivity sensors can respond to small fluctuations in sample concentration. Reproducibility can also be understood as the accuracy of the sensor. Sensors with high reproducibility can get the same results under multiple measurements. Stability is an important characteristic to ensure that the sensor can be used for a long time [45].

## 4. Application of Electrospinning in Biosensors

**Figure 5:**
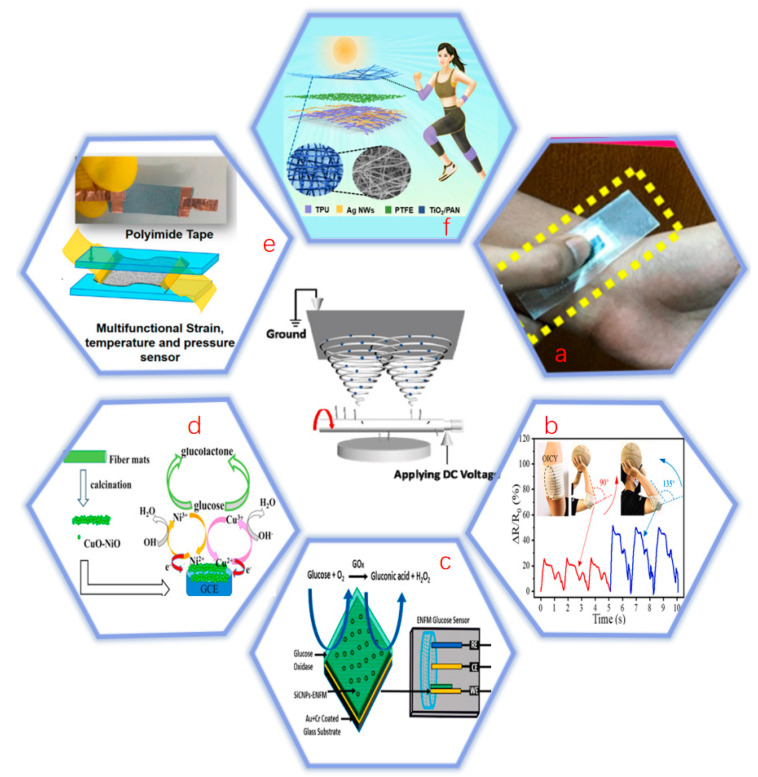
Specific applications of electrospinning-based biosensors: (**a**) monitor heart rate. Reprinted with permission from ref. [50].Copyright 2020, American Chemical Society; (**b**) monitor body movement [51]; (**c**) enzymatic biosensor for detecting glucose concentration [52]; (**d**) non-enzymatic biosensor to detect glucose concentration [53]; (**e**) detect body temperature. Reprinted with permission from ref. [54]. Copyright 2021, American Chemical Society; (**f**) detect UV intensity. Reprinted with permission from ref. [55]. Copyright 2021, American Chemical Society.

In recent years, the development of electrospinning technology has been very rapid and the fields of application are expanding. Compared with other technologies, electrospinning is relatively simple and versatile [46]. Therefore, electrospinning technology is widely used in the fields of biosensors. However, medical care has been mostly passive in recent times, with patients not seeing a doctor until they experience significant discomfort or symptoms [47]. This is very likely to cause aggravation of the disease. With the development of biosensors getting better and better, the probability of predicting the occurrence of diseases in advance is gradually increasing [48]. The biosensor can sense the biological information of the human body to determine whether there is a possibility of disease. It can also test human movement, heart rate, sweat, sleep, blood sugar, blood pressure and temperature [49]. Biosensors can also be used as a bandage. According to the principle of sensor device detection, they can be classified as: piezoelectric biosensor, electrochemical biosensor, thermal biosensor, optical biosensor, acoustic channel biosensor, field effect tube biosensor and mediator biosensor. Electrospinning technology is widely used in the first four sensors but rarely applied in the latter three. So this paper mainly introduces the first four biosensors. Figure 5 exemplifies a specific application of electrospinning-based biosensors.

### 4.1. Piezoelectric Biosensor

Piezoelectric biosensors are often used to test whether the heart rate, pulse and limb movements are normal. The detection principle is based on the deformation of the sensor to drive the resistance to change to reflect the health of the body. For example, when detecting a pulse, the resistance response is normally stable and fluctuating. However, if the resistance response suddenly appears abnormal, it means that the pulse beat is abnormal. The same is true when detecting body movement.

#### 4.1.1. Monitoring Heart Rate

Heart rate is a very important indicator of the human body and its abnormality can threaten life. Therefore, it is particularly important to invent a sensor that can monitor heart rate in real time. There are many sports bracelets on the market that can monitor heart rate in real time. However, the stability and sensitivity of this equipment are generally not high enough to reflect abnormal conditions in time [56]. This greatly limits its application in clinical medicine, and the clinical demand for low-cost and easy-to-use sensors has not yet been met [57]. The low-cost and easy operation of electrospinning greatly expands the application of this technology in the field of biosensors.

**Figure 6:**
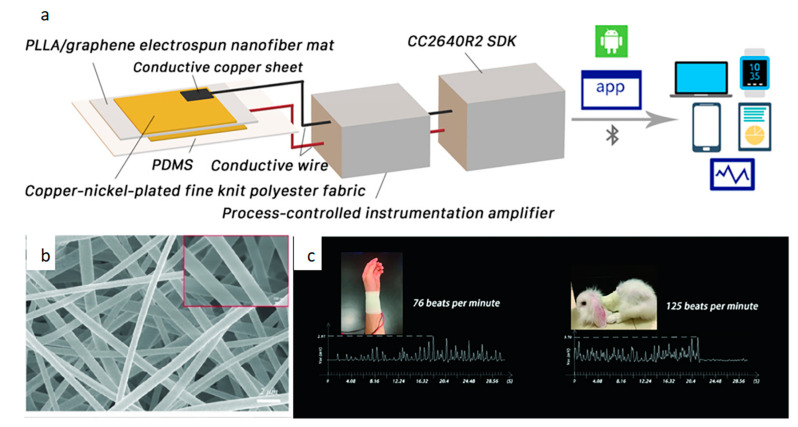
(**a**) Preparation and application of electronic skin, (**b**) SEM image of composite nanofibers, (**c**) human pulse monitoring and rabbit heart rate monitoring [60].

In recent years, the development of sensors for monitoring heart rate has advanced rapidly. Mainly manifested in increased functions, increased sensitivity and ease of production [58]. Studies have shown that many cardiovascular parameters can be obtained from heart rate, which provides a basis for early treatment of diseases [59]. Piezoelectric biosensors can respond well to heart rate conditions. The principle is to deform the dielectric in the sensor through vibration and then output a visual electrical signal. Li et al. [60] created a highly sensitive pressure sensor to monitor heart rate. Figure 6 shows the principle diagram of this sensor and the heart rate fluctuations. The electronic skin consists of Cu–Ni-plated fine-knit polyester fabric attached to both sides of the composite nanofibers to prepare the top and bottom electrodes. The electrodes are then connected via conductive copper wires. Finally, PDMS (polydimethylsiloxane) is used for external encapsulation. The electronic skin monitors pulse and heart rate changes that can be observed using smart devices. The composite nanofibers that form the core of the electronic skin show that the fibers are evenly distributed and have a uniform diameter. This allows the sensor to withstand high temperatures up to 341.0 °C. Its maximum open circuit voltage and short circuit current can reach 184.6 V and 10.8 µA, respectively. Experimental results show that it monitors the number of pulse beats consistent with normal human heart rate intervals.

#### 4.1.2. Monitoring Body Movement

The movement changes of human joints are also one of the important indicators to reflect the physical condition. Under normal circumstances, the changes of human joints are regular and do not undergo long-term mutations. However, when the changes in the joints are so abrupt that they do not stick to the same pattern, it is important to consider the health of the body to avoid getting worse. Such real-time surveillance is crucial for the early detection and treatment of disease [61]. In recent years, the piezoelectric biosensor has developed rapidly and can meet the needs of capturing abnormal human motion signals. It can also monitor movements such as pronunciation, chewing and swallowing.

**Figure 7:**
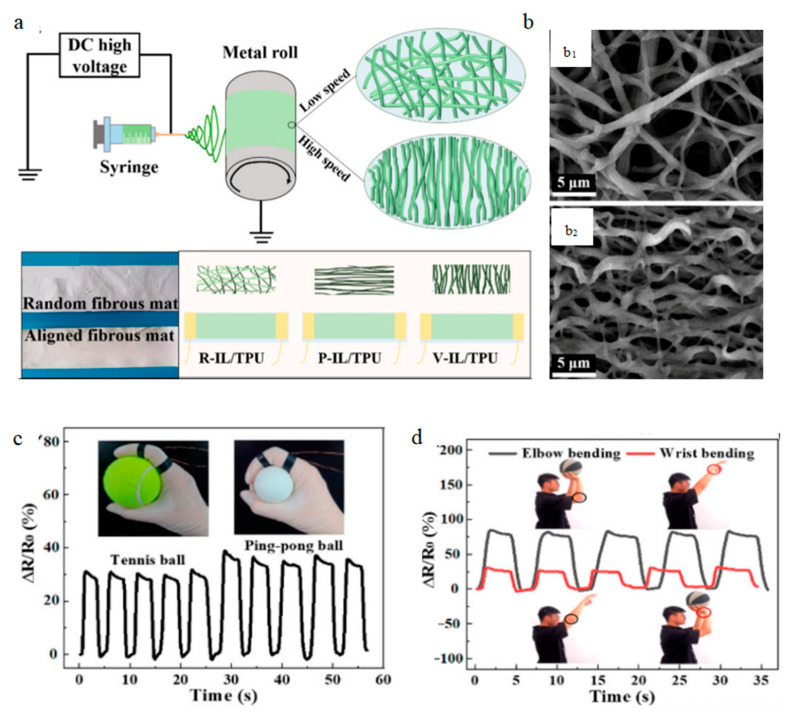
The fabrication process of the sensor and its real-time response to human motion: (**a**) the process of making the sensor; (**b**) SEM image of IL/TPU mat; (**c**) real-time response of elbow and wrist; (**d**) real-time response to finger bending. Reprinted with permission from ref. [63]. Copyright 2021, American Chemical Society.

Compared with traditional sensors, today’s sensors are more convenient and provide a more timely feedback. As time progresses, the requirements for sensors are get higher. Not only should the sensitivity and stability be high, but also the cost should be as low as possible [62]. Chen et al. [63] invented a breathable sensor to monitor human movement. Figure 7 shows the fabrication process of the sensor and its real-time response to body movement. This sensor is fabricated by shearing the prepared IL/TPU (ionic liquid/thermoplastic polyurethane) nanofiber ionogel mat in different directions and then adding electrodes at both ends. It can be seen from the figure that most of the nanofibers are aligned. This enables the sensor to have a wide response range (>200%), fast response and recovery (119 ms) and low detection limits (0.1%). It can also be used as a stretchable temperature sensor. It has high sensitivity (2.75% °C^–1^), high precision (0.1 °C) and fast response time (2.46 s).

The sensors described above are based on electrospinning. It can be seen from the data that the sensor has excellent performance and it is versatile. Table 2 exemplifies the performance comparison of electrospinning piezoelectric biosensors with other piezoelectric biosensors.

### 4.2. Electrochemical Biosensor

Electrochemical biosensors are composed of various biomolecules (enzymes, DNA, microorganisms, antibodies) and electrochemical converters (amperometric, potentiometric, capacitive and conductometric). Its main principle is to convert chemical signals into electrical signals through chemical reactions between the biological materials on the sensitive components and the biological information in the measured object. Among them, there are many sensors using enzymes as biological materials, but the disadvantage is that the activity of enzymes is difficult to guarantee. There are many sensors based on other biological materials, such as DNA, cells, nucleic acids, and microorganisms. However, due to the peculiarity of these materials, they are not widely used in the field of electrospinning. The more commonly used enzymatic biosensors and non-enzymatic biosensors are introduced here.

#### 4.2.1. Enzyme Biosensors

In recent years, the rapid development of non-invasive biosensors brings great convenience to sensor detection [74]. Human sweat, saliva and tears contain a lot of biological information. This enables the direct detection of these liquids by the enzymatic sensor. The biological information contained in these fluids can directly reflect the health of the body [75]. By detecting these liquids, you can know your physical condition more conveniently and quickly. Compared with traditional blood sampling, this method is non-invasive and cheaper. However, this method also has some disadvantages, such as the direct contact between sweat and air may lead to inaccurate measurement results.

**Figure 8:**
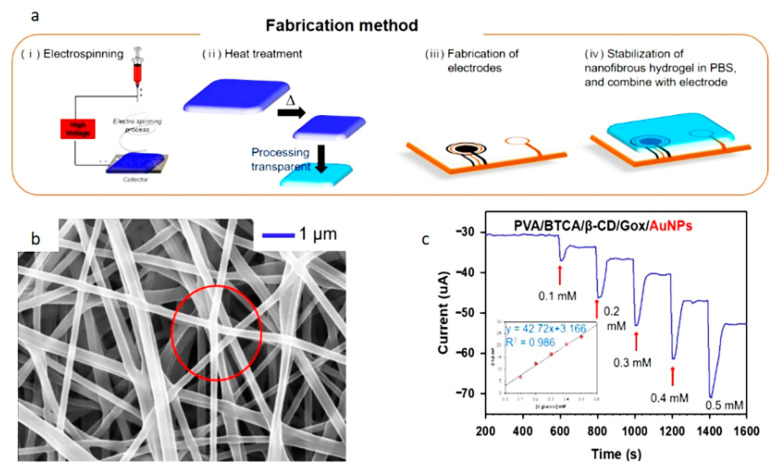
Schematic diagram of a sweat sensor: (**a**) flow chart of making hydrogel patch, (**b**) SEM image of nanofiber, (**c**) electrochemical response diagram of sensor [77].

In recent years, the main reason for the slow development of enzymatic sensors is that the inference from biological information carried in these liquids is not comprehensive enough [76]. In addition, the preservation and activity screening of enzymes are also difficult. Kim et al. [77] created a nanofiber hydrogel patch that can monitor glucose concentrations. Figure 8 shows the structure of the nanofiber hydrogel patch. The PVA NFs containing GOx/β-CD inclusion complexes with a crosslinking agent (BTCA) and AuNPs were electrospun from the aqueous solution mixture. The hydrogel was then steam treated with enzymes at 2 °C overnight. It can be seen from the figure that the fiber surface is smooth without defects. Fibers adhere but do not change shape or morphology. This gives the sensor a wide linear range (0.1 mM–0.5 mM), high sensitivity (47.2 µAmM^−1^) and low detection limit (0.01 mM).

#### 4.2.2. Non-Enzymatic Biosensors

**Figure 9:**
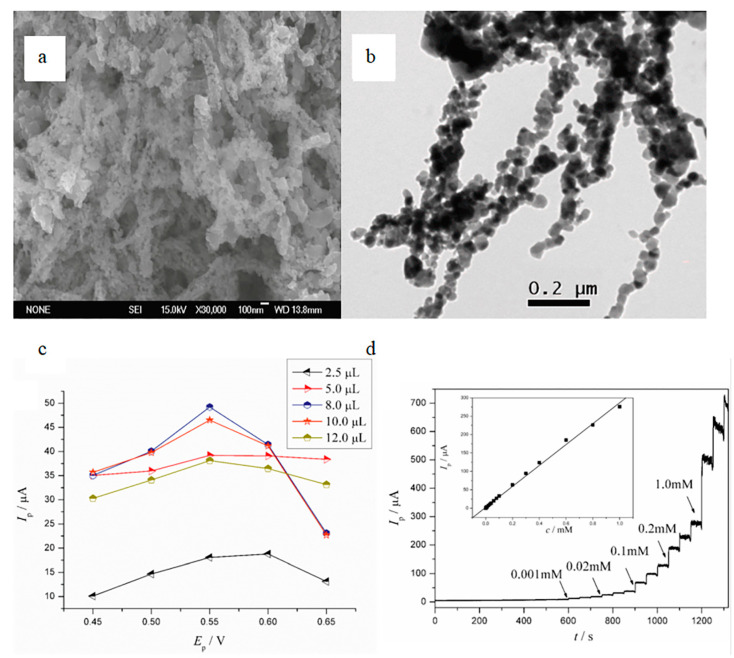
Microstructure of nanofibrous membranes and performance characterization of sensors. (**a**) SEM image of nanofiber membrane, (**b**) TEM image of nanofiber membrane, (**c**) influence of different factors on glucose oxidative current, (**d**) the current time curve of the sensor for detecting glucose [53].

The disadvantage of enzyme sensors is that enzymes are affected by various environmental factors, such as temperature, humidity, oxygen concentration and pH value. This limits the application of enzymatic sensors in continuous detection [78]. The advent of non-enzymatic biosensors avoids this disadvantage. However, compared with enzymatic sensors, the specificity of non-enzymatic sensors is poor and the cost of some sensors is relatively high. Xu et al. [53] designed a highly sensitive non-enzymatic glucose sensor based on semiconductor nanocomposites by a simple electrospinning technique. Figure 9 shows the microstructure and performance characterization of this sensor. It designs a highly sensitive non-enzymatic glucose sensor based on semiconductor nanocomposites by a simple electrospinning technique. The fabrication characteristic of this sensor is that the nanofiber membrane is calcined in a Muffle furnace for 1 h at 280 °C and then for 2 h continuously at 450 °C until the Muffle furnace is cooled to room temperature. The diameter and length of the nanofibers decreased obviously during the whole calcination process, and this special treatment of the fiber membrane makes the sensor have high sensitivity (4022 μA mM^−1^ cm^−2^) and a low detection limit (0.08 μM).

It can be seen from the above that both the enzymatic and non-enzymatic sensors have good resolution for glucose. This is because the electrospun nanofibers have a higher specific surface area and can adsorb more biological elements to fully react with the analyte. Table 3 exemplifies the performance comparison of electrospinning piezoelectric biosensors with other piezoelectric biosensors.

### 4.3. Thermosensitive Biosensors

Body temperature is one of the important signs for detecting human health [92]. The traditional way of detecting body temperature is a mercury thermometer. However, this method takes a long time and is quite difficult to operate. Later, the body temperature gun was invented which is more convenient than the traditional one.

However, the measurement results may be inaccurate compared with mercury thermometers. In recent years, temperature sensors have developed rapidly. Sensors that can feedback body temperature information in time and measure it accurately have emerged.

**Figure 10:**
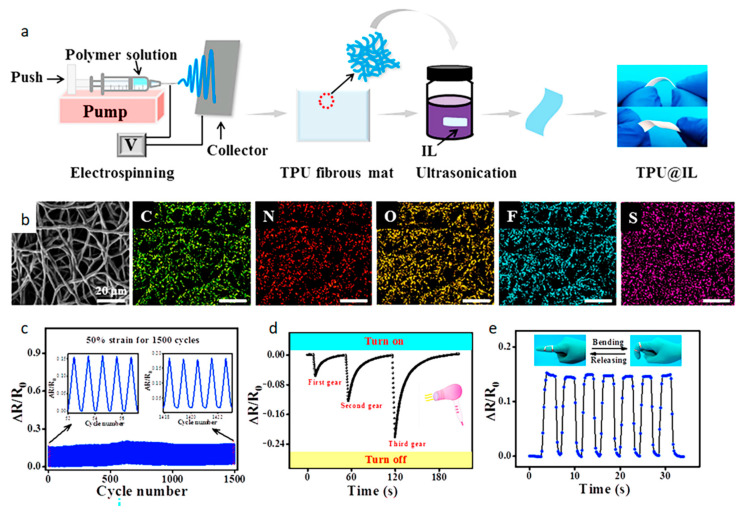
Construction and characterization of multifunctional sensor: (**a**) construction and characterization of multifunctional sensor, (**b**) fiber surface map and element map, (**c**) cyclic performance as a function of 1500 loading-unloading cycles for the sensor, (**d**) the heat flow response of the sensor to different gears of the blower, (**e**) sensor response to repeated finger bending [94].

As a wearable sensor, it must have a certain degree of biocompatibility and the material should be non-toxic [93]. The current body temperature sensors are patch type, electronic tattoo type and electronic bracelet type. These sensors are thermosensitive biosensors. Thermosensitive biosensors mainly rely on thermistors to sense temperature changes and thus lead to resistance changes. Jiang et al. [94] invented a multifunctional sensor that detects strain and temperature. Figure 10 is a manufacturing schematic and characterization of this multifunctional sensor. The sensor is made of TPU (thermoplastic polyurethane) nanofibers decorated with IL (ionic liquid) by ultrasonic anchoring technology. As can be seen from the element mapping diagram, the characteristic N, F and S elements of IL are uniformly distributed on the fiber surface. This gives the multifunctional sensor many good performances. As a strain sensor, it has a fast response time of 67 ms, ultra-low detection limit (0.1%) and ultra-wide detection range (0.1–400%). As a temperature sensor, its accuracy can reach 0.5 °C and the induction range from −40 °C to 80 °C. The sensor also provides excellent repeatability (response curve under 1500 cycles) and durability (the same signal can be obtained after 50 days).

Thermosensitive biosensors are becoming more sensitive to temperature, which results in higher requirements for sensors. Electrospun nanofibers have attracted much attention due to their excellent properties. Table 4 exemplifies the performance comparison of electrospinning thermosensitive biosensors with other thermosensitive biosensors.

### 4.4. Optical Biosensors

Humans are exposed to excessive including ultraviolet rays, which are more harmful to the human body. Long-term exposure to ultraviolet light may cause abnormalities such as skin cancer and skin aging [103]. This brings a lot of trouble to our lives. There are many ways to prevent ultraviolet radiation from having direct contact with the skin. Among them are the application of sunscreen and holding an umbrella. Wearable sensors that can detect the intensity of ultraviolet rays are relatively few. With the advancement of technology, wearable ultraviolet sensors have more and more functions. Not only can they detect the intensity of ultraviolet light, but it can also detect nearby environmental conditions. Most of these sensors use materials that are sensitive to ultraviolet light. When the UV intensity exceeds the normal level, it will cause the resistance of the sensor to change and remind the wearer to pay attention to sun protection.

**Figure 11:**
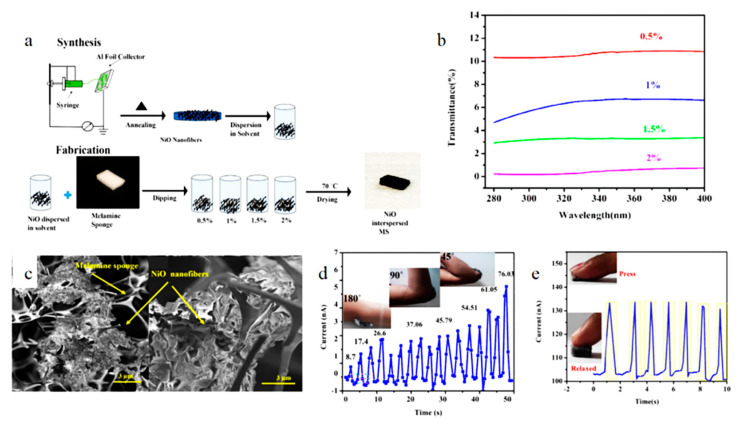
Schematic diagram and characterization of the sensor: (**a**) the production process of the sensor, (**b**) transmittance versus wavelength graph, (**c**) characterization of fiber morphology, (**d**) current response under different strains, (**e**) continuous press and relax cycles of pressure sensor. Reprinted with permission from ref. [105]. Copyright 2020, Elsevier.

Today’s wearable UV sensors are sticker-shaped, which can change color according to the intensity of UV rays [104]. They also compare the colors to get the UV intensity. Veeralingam et al. [105] invented a low-cost, tactile, high-performance and multi-functional sensing platform. Figure 11 shows the fabrication process and characterization of this sensor. The electrospun NiO nanofiber membranes were annealed in Muffle furnace for 6 h at 400 °C to decompose the polymer solution completely. Then the nanofiber membrane was uniformly dispersed in DMF (dimethylformamide) solution. A cut MS (Melamine sponge) was immersed in the solution for 1 h and then 4 h. After the fourth hour, MS was removed and dried at 70 °C for 1 h. It can be seen from the figure that, NiO fibers are interspersed evenly in the MS void. This gives the sensor numerous excellent performances. The sensor still has relatively stable current response under 500 cycles. Used for UV filter, UV protection coefficient can reach about 87.7. As a pressure sensor, the detection range is 50–700 N and the sensitivity can reach 3.75 kPa^−1^. As a strain sensor, the detection range is 7–74% and GF (gauge factor) can reach 34. Therefore, this sensor is feature-rich and has excellent performance.

Biosensors based on electrospinning to monitor light intensity are still relatively few. Electrospinning improves the performance of the sensor for light intensity detection to a certain extent. Table 5 exemplifies the performance comparison of electrospinning optical biosensors with other optical biosensors.

It can be seen from the above comparison that electrospinning technology has great advantages over other technologies. Not only does it have a better sensitivity and stability, but also its functions are broad. This is attributed to the high specific surface area of electrospun nanofibers. This also enables electrospinning technology to be widely used in the field of biosensors.

## 5. Conclusions

In recent years, electrospinning technology has developed rapidly and its application in the field of biosensors has become extensive [113]. Electrospun nanofibers have many excellent properties such as high porosity, high specific surface area, easy modification and low cost. These excellent characteristics also promote the wide application of electrospun nanofibers in the field of biosensors. Biosensors are now popular, especially in the medical field. The biosensor mainly acts on the human body to detect whether the physiological information of the human body is normal. There are many types of biosensors, which can detect various aspects of the human body’s physiological information and provide timely feedback to prevent the occurrence of diseases. It is foreseeable that the future development of electrospinning-based biosensors will have the following characteristics. (1) Diversified functions: Future biosensor functions will be more diverse and will integrate multiple functions. It can also detect physical diseases that people usually do not notice. (2) Convenience: With the advancement of nanotechnology, biosensors will become more convenient and more comfortable to wear. (3) Intelligence and integration: Future biosensors will be more closely connected to smart devices, and can form automatic collection of samples, analysis of samples and timely provide accurate results of the automated system. At the same time, chip technology will increasingly enter the field of sensors to realize the integration of detection system.

Although the development of electrospinning technology in the field of biosensors is very extensive, biosensors themselves have certain limitations that prevent them from being widely used. Because biological elements are the most important components in biosensors, biological materials on the surface of biological components are easy to inactivate and have poor reproducibility, making it difficult to guarantee their service life and storage. The biological element is closely related to the performance of the sensor. The high specific surface area of the electrospun nanofibers enables the attachment of a large number of biomaterials and contributes to the enhanced sensor performance. At the same time, electrospinning also has disadvantages, such as relatively unstable product consistency and many control factors. I believe that with the continuous advancement of electrospinning technology in future, more breakthroughs will be made in the field of biosensors.

## Figures and Tables

**Table 1:** Common biological elements and loading methods.

Biological Elements	Loading Methods	Purpose	Refs.
scherichia coli bacteriophage	electrostatic interaction	rapid detection of Escherichia coli	[39]
GOx	encapsulation of enzymes into metal frameworks for in situ growth	detect glucose	[40]
red cabbage extract (RCE)	doping in PVA solution	check pH	[41]
DNA oligonucleotides	soak the fiber membrane in the DNA solution and stir	detect p16^INK4a^ gene	[42]
burkholderia cepacia lipase (BCL)	crosslinking with nanofibers	Detect 17α- ethinylestradiol (EE2)	[43]

**Table 2:** Performance of electrospinning in detecting heart rate and pulse with other techniques.

Methods	Main Material	Sensitivity	Linear Range	Stability	Refs.
Electrospinning	PVDF	18.376 kPa^−1^	0.002–10 kPa	7500	[64]
	PVDF	0.38 V/N	— —	6834	[65]
	PVDF	5 kPa^−1^	0–5 kPa	— —	[66]
	CA	60.28 kPa^−1^	0–24 kPa	13,000	[67]
	P(VDF-TrFE)	437.5 mV/μm 41.7 mV/μm	0–2 μm 2–10 μm	2000	[68]
Fiber Optic	POF	0.002 mV/μm 0.0004 mV/μm	150–650 μm 1400–3450 μm	— —	[69]
Sacrifice template and sandpaper-treated	PDMS	39.077 kPa^−1^	0.0009–160 kPa	1400	[70]
Laser etching	PVDF	0.24 V/N	— —	4000	[71]
Coating	PU	4.169 kPa^−1^	0.02–10.3 kPa	2300	[72]
	TPU	1.02 kPa^−1^	0.0007–160 kPa	60,000	[73]

**Table 3:** Performance comparison of electrospinning and other technologies in detecting body fluids.

Methods	Main Material	Sensitivity	Linear Range	Stability	Refs.
Electrospinning	PAN	301.77 μAmM^−1^ cm^−2^	0.0003–4.5 mM	80 days	[79]
	PAN	1947.2 μAmM^−1^ cm^−2^	0.005–19.175 mM	— —	[80]
Coating	PVDF	5.18 μAmM^−1^ cm^−2^	— —	— —	[81]
	fabric	105.93 μAmM^−1^ cm^−2^	0.05–1 mM	— —	[82]
	PU	12.69 μAmM^−1^ cm^−2^	1–30 mM	16 days	[83]
Hydrogel	PEDOT:PSS	0.875 µAµM^−1^ cm^−2^	2.0–250 μmolL^–1^	25 days	[84]
Chemical vapor deposition	nickel textile	14.45 µAµM^−1^ cm^−2^	— —	— —	[85]
Wet spinning	PU	140 μAmM^−1^ cm^−2^	— —	10,000	[86]
	PU	425.9 μAmM^−1^ cm^−2^	10 μM–0.66 mM	— —	[87]
	Ni(OH)	595.3 μAmM^−1^ cm^−2^	0.01–7.66 mM	— —	[88]
Deposition	PDMS	253.4 μAmM^−1^ cm^−2^	— —	1000	[89]
In-situ synthesized	fabric	1625 μAmM^−1^ cm^−2^	0.001–1 mM	— —	[90]
		1325 μAmM^−1^ cm^−2^	1–10 mM		
Dry Spinning	SEBS	11.7 μAmM^−1^ cm^−2^	0–500 μM	— —	[91]

**Table 4:** Performance of electrospinning for body temperature detection with other technologies.

Methods	Main Material	Sensitivity	Linear Range	Stability	Refs.
Electrospinning	PVDF TPU	57.76/°C 2.75%/°C	24–48 °C — —	5000 1000	[54] [63]
Fiber Optic	PANI	8.962 nm/°C	33–43 °C	— —	[93]
	PDMS	1.3%/°C	20–50 °C	— —	[95]
Electrochemical Deposition	GuHCl	1.75%/°C	35–63 °C	— —	[96]
Wet-spinning	PU	0.8%/°C	— —	— —	[97]
Spray-coated	AgNW	0.47 Ω/°C	25–60 °C	1000	[98]
	PEDOT:PSS	−0.99%/°C	20–50 °C	— —	[99]
Free radical polymerization	NIPAAm	−1.39%/°C	30–37 °C	— —	[100]
		0.37%/°C	37–43 °C		
In situ synthesized	TPU	0.95%/°C	20–40 °C	— —	[101]
Drop coating	PEDOT:PSS	−0.803%/°C	35–40 °C	— —	[102]

**Table 5:** Performance comparison of electrospinning applied to detect UV and other technologies.

Methods	Main Material	Sensitivity	Linear Range	Stability	Refs.
Electrospinning	PAN	0.0574%	— —	— —	[55]
	TPU	7.2%	— —	— —	[106]
Fiber-Optic	ZnO ZnO	7.096 W/(mWcm^−^^2^) — —	1527–1534 nm 1.5–12.5 mWcm^−^^2^	— — — —	[107] [108]
Spin-coated	BaTiO_3_	23.11% 46.85% 67.92%	0.07 mWcm^−^^2^ 0.3 mWcm^−^^2^ 1.1 mWcm^−^^2^	— —	[109]
Rf-sputtering	ZnO	50 µW/cm^2^	— —	100	[110]
Hydrogels	PVA	10.88%	365 nm	— —	[111]
		78.26%	650 nm		
Ink-coating	Cellulose thread	4.76 mW/cm^2^	254 nm	— —	[112]
		0.76% mW/cm^2^	365 nm		

## Data Availability

Not applicable.

## References

[1] Lakshmanan A., Jin Z., Nety S.P., Sawyer D.P., Gosselin A.L., Malounda D., Swift M.B., Maresca D., Shapiro M.G. (2020). Publisher Correction: Acoustic biosensors for ultrasound imaging of enzyme activity. Nat. Chem. Biol..

[2] Zhang P., Zhao X., Zhang X., Lai Y., Wang X., Li J., Wei G., Su Z. (2014). Electrospun Doping of Carbon Nanotubes and Platinum Nanoparticles into the β-Phase Polyvinylidene Difluoride Nanofibrous Membrane for Biosensor and Catalysis Applications. ACS Appl. Mater. Interfaces.

[3] Yang T., Zhan L., Huang C.Z. (2020). Recent insights into functionalized electrospun nanofibrous films for chemo-/bio-sensors. TrAC Trends Anal. Chem..

[4] Arida I.A., Ali I.H., Nasr M., El-Sherbinyet I.M. (2021). Electrospun polymer-based nanofiber scaffolds for skin regeneration. J. Drug Deliv. Sci. Technol..

[5] Senthamizhan A., Balusamy B., Uyar T. (2020). Recent progress on designing electrospun nanofibers for colorimetric biosensing applications. Curr. Opin. Biomed. Eng..

[6] Liu X., Cheng H., Zhao Y., Wang Y., Li F. (2022). Portable electrochemical biosensor based on laser-induced graphene and MnO2 switch-bridged DNA signal amplification for sensitive detection of pesticide. Biosens. Bioelectron..

[7] Wang L., Sipe D.M., Xu Y., Lin Q. (2008). A MEMS Thermal Biosensor for Metabolic Monitoring Applications. J. Microelectromech. Syst..

[8] Oh J., Yoo G., Chang Y.W., Kim H.J., Jose J., Kim E., Pyun J., Yoo K.H. (2013). A carbon nanotube metal semiconductor field effect transistor-based biosensor for detection of amyloid-beta in human serum. Biosens. Bioelectron..

[9] Jain S., Paliwal A., Gupta V., Tomar M. (2022). Smartphone integrated handheld Long Range Surface Plasmon Resonance based fiber-optic biosensor with tunable SiO2 sensing matrix. Biosens. Bioelectron..

[10] Jandas P.J., Luo J.T., Prabakaran K., Chen F., Fu Y.Q. (2020). Highly stable, love-mode surface acoustic wave biosensor using Au nanoparticle-MoS2-rGO nano-cluster doped polyimide nanocomposite for the selective detection of carcinoembryonic antigen. Mater. Chem. Phys..

[11] Li C., Li Z., Suo S., Li X., Cheng Q., Meng S. (2021). Sensitivity enhancement by employing BiFeO3 and graphene hybrid structure in surface plasmon resonance biosensors. Opt. Mater..

[12] Hong C.-C., Lin C.C., Hong C.L., Lin Z.X., Chung M.H., Hsieh P.W. (2016). Handheld analyzer with on-chip molecularly-imprinted biosensors for electrical detection of propofol in plasma samples. Biosens. Bioelectron..

[13] Moore E., Rawley O., Wood T., Galvin P. (2009). Monitoring of cell growth in vitro using biochips packaged with indium tin oxide sensors. Sens. Actuators B Chem..

[14] Carrascosa L.G., Huertas C.S., Lechuga L.M. (2016). Prospects of optical biosensors for emerging label-free RNA analysis. TrAC Trends Anal. Chem..

[15] Jabbari Behrouz S., Rahmani O., Hosseini S.A. (2019). On nonlinear forced vibration of nano cantilever-based biosensor via couple stress theory. Mech. Syst. Signal Process..

[16] Farmani H., Farmani A., Nguyen T.A., Thomas S., Ahmadi M., Joshi N., Nguyen T.A., Yasin G. (2022). 12-Graphene-based field effect transistor (GFET) as nanobiosensors. Silicon-Based Hybrid Nanoparticles.

[17] Nuzaihan M.N.M., Hashim U., Md Arshad M.K., Kasjoo S.R., Rahman S.F.A., Ruslinda A.R., Fathil M.F.M., Adzhri R., Shahimin M.M. (2016). Electrical detection of dengue virus (DENV) DNA oligomer using silicon nanowire biosensor with novel molecular gate control. Biosens. Bioelectron..

[18] Song Y., Min J., Yu Y., Wang H., Gao W. (2020). Wireless battery-free wearable sweat sensor powered by human motion. Sci. Adv..

[19] Peng X., Dong K., Ye C., Jiang Y., Wang Z.L. (2020). A breathable, biodegradable, antibacterial, and self-powered electronic skin based on all-nanofiber triboelectric nanogenerators. Sci. Adv..

[20] Cordeiro C.A., Vries M.D., Ngabi W., Oomen P.E., Gremers T.I., Westerink B.H.C. (2015). In vivo continuous and simultaneous monitoring of brain energy substrates with a multiplex amperometric enzyme-based biosensor device. Biosens. Bioelectron..

[21] Smoak M.M., Hogan K.J., Grande-Allen K.J., Mikos A.G. (2021). Bioinspired electrospun dECM scaffolds guide cell growth and control the formation of myotubes. Sci. Adv..

[22] Guo J., Fu S., Deng Y., Xu X., Laima S., Liu D., Zhang P., Zhou J., Zhao H., Yu H. (2022). Hypocrystalline ceramic aerogels for thermal insulation at extreme conditions. Nature.

[23] Cheng X., Liu Y., Si Y., Yu J., Ding B. (2022). Direct synthesis of highly stretchable ceramic nanofibrous aerogels via 3D reaction electrospinning. Nat. Commun..

[24] Singh R.K., Lye S.W., Miao J. (2021). Holistic investigation of the electrospinning parameters for high percentage of β-phase in PVDF nanofibers. Polymer.

[25] Gade H., Nikam S., Chase G.G., Reneker D.H. (2021). Effect of electrospinning conditions on β-phase and surface charge potential of PVDF fibers. Polymer.

[26] Raksa A., Numpaisal P.O., Ruksakulpiwat Y. (2021). The effect of humidity during electrospinning on morphology and mechanical properties of SF/PVA nanofibers. Mater. Today: Proc..

[27] Raman A., Jayan J.S., Bds D., Appukuttan S., Joseph K. (2021). Electrospun Nanofibers as Effective Superhydrophobic Surfaces: A Brief review. Surf. Interfaces.

[28] Liu Y., Hao M., Chen Z., Liu L., Liu Y., Yang W., Ramakrishna S. (2020). A review on recent advances in application of electrospun nanofiber materials as biosensors. Curr. Opin. Biomed. Eng..

[29] Bayan M.A.H., Taromi F.A., Lanzi M., Pierini F. (2021). Enhanced efficiency in hollow core electrospun nanofiber-based organic solar cells. Sci. Rep..

[30] Yang G., Tang X., Zhao G., Li Y., Ma C., Zhuang X., Yan J. (2022). Highly sensitive, direction-aware, and transparent strain sensor based on oriented electrospun nanofibers for wearable electronic applications. Chem. Eng. J..

[31] Farboudi A., Mahboobnia K., Chogan F., Karimi M., Askari A., Banihashem S., Davaran S., Irani M. (2020). UiO-66 metal organic framework nanoparticles loaded carboxymethyl chitosan/poly ethylene oxide/polyurethane core-shell nanofibers for controlled release of doxorubicin and folic acid. Int. J. Biol. Macromol..

[32] Zia Q., Tabassum M., Lu Z., Khawar M.T., Li J. (2020). Porous poly(L–lactic acid)/chitosan nanofibres for copper ion adsorption. Carbohydr. Polym..

[33] Fu Y., Cheng Y., Chen C., Li D., Zhang W. (2022). Study on preparation process and enhanced piezoelectric performance of pine-needle-like ZnO@PVDF composite nanofibers. Polym. Test..

[34] Zhang K., Zhao W., Liu Q., Yu M. (2021). A new magnetic melt spinning device for patterned nanofiber. Sci. Rep..

[35] Wu J., Qin X., Miao C., He Y.B., Liang G., Zhou D., Liu M., Han C., Li B., Kang F. (2016). A honeycomb-cobweb inspired hierarchical core–shell structure design for electrospun silicon/carbon fibers as lithium-ion battery anodes. Carbon.

[36] Zou D., Wang W., Liu J., Weng J., Duan J., Zhou J., Zhou P. (2022). Insights into the storage mechanism of novel mesoporous hollow TiO2-x/C nanofibers as a high-performance anode material for sodium-ion batteries. Carbon.

[37] Kadadou D., Tizani L., SWadi V., Banat F., Alsafar H., FYousef A., Barcelo D., WHasan S. (2022). Recent advances in the biosensors application for the detection of bacteria and viruses in wastewater. J. Environ. Chem. Eng..

[38] Jiménez-Rodríguez M.G., Silva-Lance F., Parra-Arroyo L., Medina-Salazar D.A., Martínez-Ruiz M., Melchor-Martínez E.M., Martínez-Prado M.A., Iqbal H., Parra-Saldívar R., Barceló D. (2022). Biosensors for the detection of disease outbreaks through wastewater-based epidemiology. TrAC Trends Anal. Chem..

[39] Wang F.R., Wang R. (2022). Modification of polyacrylonitrile-derived carbon nanofibers and bacteriophages on screen-printed electrodes: A portable electrochemical biosensor for rapid detection of Escherichia coli. Bioelectrochemistry.

[40] Li X., Feng Q., Lu K., Huang J., Zhang Y., Hou Y., Qiao H., Li D., Wei Q. (2021). Encapsulating enzyme into metal-organic framework during in-situ growth on cellulose acetate nanofibers as self-powered glucose biosensor. Biosens. Bioelectron..

[41] Maftoonazad N., Ramaswamy H. (2019). Design and testing of an electrospun nanofiber mat as a pH biosensor and monitor the pH associated quality in fresh date fruit (Rutab). Polym. Test..

[42] Wang X., Wang Y., Shan Y., Jiang M., Jin X., Gong M., Xu J. (2018). A novel and sensitive electrogenerated chemiluminescence biosensor for detection of p16INK4a gene based on the functional paste-like nanofibers composites-modified screen-printed carbon electrode. J. Electroanal. Chem..

[43] Pavinatto A., Mercante L.A., Facure Murilo H.M., Pena R.B., Sanfelice R.C., Luiz H.C., Correa D.S. (2018). Ultrasensitive biosensor based on polyvinylpyrrolidone/chitosan/reduced graphene oxide electrospun nanofibers for 17α—Ethinylestradiol electrochemical detection. Appl. Surf. Sci..

[44] Chadha U., Bhardwaj P., Rawat P., Agarwal R., Gupta I., Panjwani M., Singh S., Ahuja C., Selvaraj S.K., Banavoth M. (2022). Recent progress and growth in biosensors technology: A critical review. J. Ind. Eng. Chem..

[45] Zhu P., Peng H., Rwei A.Y. (2022). Flexible, wearable biosensors for digital health. Med. Nov. Technol. Devices.

[46] Wu J., Yin F. (2013). Sensitive enzymatic glucose biosensor fabricated by electrospinning composite nanofibers and electrodepositing Prussian blue film. J. Electroanal. Chem..

[47] Wei X., Zhu M., Li J., Liu L., Ding B. (2021). Wearable biosensor for sensitive detection of uric acid in artificial sweat enabled by a fiber structured sensing interface. Nano Energy.

[48] Yue Y., Gong X., Jiao W., Li Y., Ding B. (2021). In-situ electrospinning of thymol-loaded polyurethane fibrous membranes for waterproof, breathable, and antibacterial wound dressing application. J. Colloid Interface Sci..

[49] Luraghi A., Peri F., Moroni L. (2021). Electrospinning for drug delivery applications: A review. J. Control. Release.

[50] Maity K., Garain S., Henkel K., Schmeier D., Mandal D. (2020). Self-Powered Human-Health Monitoring through Aligned PVDF Nanofibers Interfaced Skin-Interactive Piezoelectric Sensor. ACS Appl. Polym. Mater..

[51] Tang J., Wu Y., Ma S., Yan T., Pan Z. (2022). Flexible strain sensor based on CNT/TPU composite nanofiber yarn for smart sports bandage. Compos. Part B Eng..

[52] Puttananjegowda K., Takshi A., Thomas S. (2021). Silicon carbide nanoparticles electrospun nanofibrous enzymatic glucose sensor. Biosens. Bioelectron..

[53] Xu Y., Ding Y., Zhang L., Zhang X. (2021). Highly sensitive enzyme-free glucose sensor based on CuO–NiO nanocomposites by electrospinning. Compos. Commun..

[54] Veeralingam S., Badhulika S. (2021). Bi2S3/PVDF/Ppy-Based Freestanding, Wearable, Transient Nanomembrane for Ultrasensitive Pressure, Strain, and Temperature Sensing. ACS Appl. Bio Mater..

[55] Jiang Y., Dong K., An J., Liang F., Yi J., Peng X., Ning C., Ye C., Wang Z. (2021). UV-Protective, Self-Cleaning, and Antibacterial Nanofiber-Based Triboelectric Nanogenerators for Self-Powered Human Motion Monitoring. ACS Appl. Mater. Interfaces.

[56] Xiao N., Yu W., Han X. (2020). Wearable heart rate monitoring intelligent sports bracelet based on Internet of things. Measurement.

[57] Kaisti M., Panula T., Leppanen J., Punkkinen R., Tadi M.J., Vasankari T., Jaakkola S., Kivinieimi T., Airaksinen J., Kostiainen P. (2019). Clinical assessment of a non-invasive wearable MEMS pressure sensor array for monitoring of arterial pulse waveform, heart rate and detection of atrial fibrillation. Npj Digit. Med..

[58] Shahrestani S., Chou T.C., Shang K.M., Zada G., Borok Z., PRao A., Tai Y.C. (2021). A wearable eddy current based pulmonary function sensor for continuous non-contact point-of-care monitoring during the COVID-19 pandemic. Sci. Rep..

[59] Lin J., Fu R., Zhong X., Yu P., Tan G., Li W. (2021). Wearable sensors and devices for real-time cardiovascular disease monitoring. Cell Rep. Phys. Sci..

[60] Li X., Chen S., Zhang X., Li J., Liu H., Han N. (2020). Poly-l-Lactic Acid/Graphene Electrospun Composite Nanofibers for Wearable Sensors. Energy Technol..

[61] Nasiri S., Khosravani M.R. (2020). Progress and challenges in fabrication of wearable sensors for health monitoring. Sens. Actuators A Phys..

[62] Qi K., He J., Wang H., Zhou Y., Cui S. (2017). A Highly Stretchable Nanofiber-Based Electronic Skin with Pressure-, Strain-, and Flexion-Sensitive Properties for Health and Motion Monitoring. ACS Appl. Mater. Interfaces.

[63] Chen J., Wang F., Zhu G., Wang C., Cui X., Xi M., Chang X., Zhu Y. (2021). Breathable Strain/Temperature Sensor Based on Fibrous Networks of Ionogels Capable of Monitoring Human Motion, Respiration, and Proximity. ACS Appl. Mater. Interfaces.

[64] Zhou Y., He J., Wang H., Qi K., Nan N., You X., Shao W., Wang L., Ding B., Cui S. (2017). Highly sensitive, self-powered and wearable electronic skin based on pressure-sensitive nanofiber woven fabric sensor. Sci. Rep..

[65] Su Y., Li W., Yuan L., Chen C., Pan H., Xie G., Conta G., Ferrier S., Zhao X., Chen G. (2021). Piezoelectric fiber composites with polydopamine interfacial layer for self-powered wearable biomonitoring. Nano Energy.

[66] Yang C.-R., Lin M.F., Huang C.K., Huang W.C., Tseng S.F., Chiang H.H. (2022). Highly sensitive and wearable capacitive pressure sensors based on PVDF/BaTiO3 composite fibers on PDMS microcylindrical structures. Measurement.

[67] Qin Z., Lv Y., Fang X., Zhao B., Niu F., Min L., Pan K. (2022). Ultralight polypyrrole crosslinked nanofiber aerogel for highly sensitive piezoresistive sensor. Chem. Eng. J..

[68] Kang S., Kim S.H., Lee H.B., Mhin S., Ryu J.H., Kim Y.W., LJones J., Son Y., Lee N.K., Lee K. (2022). High-power energy harvesting and imperceptible pulse sensing through peapod-inspired hierarchically designed piezoelectric nanofibers. Nano Energy.

[69] Yhuwana Y.G.Y., Apsari R., Yasin M. (2017). Fiber optic sensor for heart rate detection. Optik.

[70] Ni Y., Liu L., Huang J., Li S., Chen Z., Zhang W., Lai Y. (2022). Rational designed microstructure pressure sensors with highly sensitive and wide detection range performance. J. Mater. Sci. Technol..

[71] Yuan Y., Chen H., Xu H., Jin Y., Chen G., Zheng W., Wang W., Wang Y., Gao L. (2022). Highly sensitive and wearable bionic piezoelectric sensor for human respiratory monitoring. Sens. Actuators A Phys..

[72] Zhu G., Ren P.G., Wang J., Duan Q., Yan D.X. (2020). A Highly Sensitive and Broad-Range Pressure Sensor Based on Polyurethane Mesodome Arrays Embedded with Silver Nanowires. ACS Appl. Mater. Interfaces.

[73] Yin Y.M., Li H.Y., Xu J., Zhang C., Zhu G. (2021). Facile Fabrication of Flexible Pressure Sensor with Programmable Lattice Structure. ACS Appl. Mater. Interfaces.

[74] Poletti F., Zanfrognini B., Favaretto L., Quintano V., Sun J., Treossi E., Melucci M., Palermo V., Zanardi C. (2021). Continuous capillary-flow sensing of glucose and lactate in sweat with an electrochemical sensor based on functionalized graphene oxide. Sens. Actuators B Chem..

[75] Zhou J., Men D., Zhang X.-E. (2022). Progress in wearable sweat sensors and their applications. Chin. J. Anal. Chem..

[76] Nyein H., Bariya M., Kivimki L., Uusitalo S., Javey A. (2019). Regional and correlative sweat analysis using high-throughput microfluidic sensing patches toward decoding sweat. Sci. Adv..

[77] Kim G.J., Kim K.O. (2020). Novel glucose-responsive of the transparent nanofiber hydrogel patches as a wearable biosensor via electrospinning. Sci. Rep..

[78] Taşaltın C. (2021). Glucose sensing performance of PAN: β-rhombohedral borophene based non-enzymatic electrochemical biosensor. Inorg. Chem. Commun..

[79] Rani S.D., Ramachandran R., Sheet S., Aziz M.A., Kumar G.G. (2020). NiMoO4 nanoparticles decorated carbon nanofiber membranes for the flexible and high performance glucose sensors. Sens. Actuators B Chem..

[80] Liu L., Wang Z., Yang J., Liu G., Li J., Guo L., Shen S., Guo Q. (2018). NiCo2O4 nanoneedle-decorated electrospun carbon nanofiber nanohybrids for sensitive non-enzymatic glucose sensors. Sens. Actuators B Chem..

[81] Kanokpaka P., Chang L.Y., Wang B.C., Huang T.H., Shih M.J., Hung W.S., Lai J.Y., Ho K.C., Yeh M.H. (2022). Self-powered molecular imprinted polymers-based triboelectric sensor for noninvasive monitoring lactate levels in human sweat. Nano Energy.

[82] Zheng L., Liu Y., Zhang C. (2021). A sample-to-answer, wearable cloth-based electrochemical sensor (WCECS) for point-of-care detection of glucose in sweat. Sens. Actuators B Chem..

[83] Jin X., Li G., Xu T., Su L., Yan D., Zhang X. (2022). Fully integrated flexible biosensor for wearable continuous glucose monitoring. Biosens. Bioelectron..

[84] Xu Z., Song J., Liu B., Lv S., Gao F., Luo X., Wang P. (2021). A conducting polymer PEDOT:PSS hydrogel based wearable sensor for accurate uric acid detection in human sweat. Sens. Actuators B Chem..

[85] Yao Y., Chen J., Guo Y., Lv T., Chen T. (2021). Integration of interstitial fluid extraction and glucose detection in one device for wearable non-invasive blood glucose sensors. Biosens. Bioelectron..

[86] Toi P.T., Trung T.Q., Dang T., Bae C.W., Lee N.E. (2019). Highly Electrocatalytic, Durable, and Stretchable Nanohybrid Fiber for On-Body Sweat Glucose Detection. ACS Appl. Mater. Interfaces.

[87] Shu Y., Su T., Lu Q., Shang Z., Xu Q., Hu X. (2021). Highly Stretchable Wearable Electrochemical Sensor Based on Ni-Co MOF Nanosheet-Decorated Ag/rGO/PU Fiber for Continuous Sweat Glucose Detection. Anal. Chem..

[88] Zhu Z., Wu Y., Yang J., Xue Y. (2021). Core-sheath fibers composed of F-doped nickel hydroxide nanorods and graphene fibers for effective fiber-shaped nonenzymatic glucose sensors. J. Alloys Compd..

[89] Bae C.W., Toi P.T., Kim B.Y., Lee W.I., Lee H.B., Hanif A., Lee E.H., Lee N.E. (2019). Fully Stretchable Capillary Microfluidics-Integrated Nanoporous Gold Electrochemical Sensor for Wearable Continuous Glucose Monitoring. ACS Appl. Mater. Interfaces.

[90] Sedighi A., Montazer M., Mazinani S. (2019). Synthesis of wearable and flexible NiP0.1-SnOx/PANI/CuO/cotton towards a non-enzymatic glucose sensor. Biosens. Bioelectron..

[91] Zhao Y., Zhai Q., Dong D., An T., Gong S. (2019). Highly Stretchable and Strain-Insensitive Fiber-Based Wearable Electrochemical Biosensor to Monitor Glucose in the Sweat. Anal. Chem..

[92] Han S., Kim J., Won S.M., Ma Y., Kang D., Xie Z., Lee K., Chung H.U., Banks A., Min S. (2018). Battery-free, wireless sensors for full-body pressure and temperature mapping. Sci. Transl. Med..

[93] Wang S.N., Lv R.Q., Zhao Y., Qian J.K. (2018). A Mach-Zehnder interferometer-based High Sensitivity Temperature sensor for human body monitoring. Opt. Fiber Technol..

[94] Jiang N., Li H., Hu D., Xu Y., Hu Y., Zhu Y., Han X., Zhao G., Chen J., Chang X. (2021). Stretchable strain and temperature sensor based on fibrous polyurethane film saturated with ionic liquid. Compos. Commun..

[95] Chen M., He Y., Liang H., Zhou H., Wang X., Heng X., Zhang Z., Gan J., Yang Z. (2022). Stretchable and Strain-Decoupled Fluorescent Optical Fiber Sensor for Body Temperature and Movement Monitoring. ACS Photonics.

[96] Yang Z., Huang T., Cao P., Cui Y., Nie J., Chen T., Yang H., Wang F., Sun L. (2022). Carbonized Silk Nanofibers in Biodegradable, Flexible Temperature Sensors for Extracellular Environments. ACS Appl. Mater. Interfaces.

[97] Trung T.Q., Dang T., Ramasundaram S., Toi P.T., Lee N.E. (2019). A Stretchable Strain-Insensitive Temperature Sensor Based on Free-Standing Elastomeric Composite Fibers for On-Body Monitoring of Skin Temperature. ACS Appl. Mater. Interfaces.

[98] Cui Z., Poblete F.R., Zhu Y. (2019). Tailoring the Temperature Coefficient of Resistance of Silver Nanowire Nanocomposites and their Application as Stretchable Temperature Sensors. ACS Appl. Mater. Interfaces.

[99] Pradhan S., Yadavalli V.K. (2021). Photolithographically Printed Flexible Silk/PEDOT:PSS Temperature Sensors. ACS Appl. Electron. Mater..

[100] Pang Q., Hu H., Zhang H., Qiao B., Ma L. (2022). Temperature-Responsive Ionic Conductive Hydrogel for Strain and Temperature Sensors. ACS Appl. Mater. Interfaces.

[101] Li F., Xue H., Lin X., Zhao H., Zhang T. (2022). Wearable Temperature Sensor with High Resolution for Skin Temperature Monitoring. ACS Appl. Mater. Interfaces.

[102] Song J., Wei Y., Xu M., Gao J., Luo L., Wu H., Li X., Li Y., Wang X. (2022). Highly Sensitive Flexible Temperature Sensor Made Using PEDOT:PSS/PANI. ACS Appl. Polym. Mater..

[103] Pielak R., Wei P., Peyret H., Balooch G., Rogers J. (2020). 14188 Wearable UV/HEV light sensor and smartphone application for personal monitoring and personalized recommendations. J. Am. Acad. Dermatol..

[104] Araki H., Kim J., Zhang S., Banks A., Crawford K.E., Sheng X., Gutruf P., Shi Y., Pielak R.M., Rogers J.A. (2017). Materials and Device Designs for an Epidermal UV Colorimetric Dosimeter with Near Field Communication Capabilities. Adv. Funct. Mater..

[105] Veeralingam S., Priya S., Badhulika S. (2020). NiO nanofibers interspersed sponge based low cost, multifunctional platform for broadband UV protection, ultrasensitive strain and robust finger-tip skin inspired pressure sensor. Chem. Eng. J..

[106] Cui X., Chen J., Wu W., Liu Y., Li H., Xu Z., Zhu Y. (2022). Flexible and breathable all-nanofiber iontronic pressure sensors with ultraviolet shielding and antibacterial performances for wearable electronics. Nano Energy.

[107] Feng Y., Shen T., Li X., Wei X. (2020). ZnO-nanorod–fiber UV sensor based on evanescent field principle. Optik.

[108] Yu Z., Xu J., Gong H. (2022). Bioinspired Self-Powered Piezoresistive Sensors for Simultaneous Monitoring of Human Health and Outdoor UV Light Intensity. ACS Appl. Mater. Interfaces.

[109] Xu Q., Lou J., Zhang R., Ma B., Qin Y. (2020). Self-Cleaning and Self-Powered UV Sensors for Highly Reliable Outdoor UV Detection. ACS Appl. Electron. Mater..

[110] Sahare P.D., Srivastava S.K., Surender K., Fouran S. (2018). n-ZnO/p-Si heterojunction nanodiodes based sensor for monitoring UV radiation. Sens. Actuators A Phys..

[111] Yin J., Lu C., Li C., Yu Z., Shen C., Yang Y., Jiang X., Zhang Y. (2022). A UV-filtering, environmentally stable, healable and recyclable ionic hydrogel towards multifunctional flexible strain sensor. Compos. Part B Eng..

[112] Kim S.J., Moon D.I., Seol M.L., Kim B., Han J.W., Meyyappan M. (2018). Wearable UV Sensor Based on Carbon Nanotube-Coated Cotton Thread. ACS Appl. Mater. Interfaces.

[113] Chen Y.W., Yu Q.W., Chen C., Zhang W.W., Yu C., Yang C.H., Jin S.X., Xin B.J. Research of Polysulfone Amide Micronanofiber Forming Technology. Proceedings of the Textile Bioengineering and Informatics Symposium Proceedings (TBIS 2017).

